# Overexpression of HES6 has prognostic value and promotes metastasis via the Wnt/β-catenin signaling pathway in colorectal cancer

**DOI:** 10.3892/or.2022.8309

**Published:** 2022-03-23

**Authors:** Yuandong Xu, Xuejuan Liu, Huizhong Zhang, Ziyuan Zhu, Xianqiu Wu, Xiaobing Wu, Shuling Li, Libing Song, Xuehu Xu

Oncol Rep 40: 1261–1274, 2018; DOI: 10.3892/or.2018.6539

Subsequently to the publication of the above article, an interested reader drew to the authors attention that Fig. 2 on p. 1266 and Fig. 5 on p. 1269 contained some apparent errors in terms of the assembly of the various data panels. Specifically, Fig. 2D appeared to contain a pair of overlapping images, and Figs. 5D and 8A also appeared to include overlapping images. However, the authors were able to consult their original data, and assess where the errors had been made during the compilation of these figures.

The corrected versions of Figs. 2 (showing the correct data for the ‘5T’ panel in Fig. 2D) and 5 (showing alternative data) are shown on the subsequent pages. The authors regret the errors that were made during the preparation of the published figures, and confirm that these errors did not grossly affect the conclusions reported in the study. The authors are grateful to the Editor of *Oncology Reports* for allowing them the opportunity to publish a Corrigendum, and all the authors agree to this Corrigendum. Furthermore, they apologize to the readership for any inconvenience caused.

## Figures and Tables

**Figure 2.**
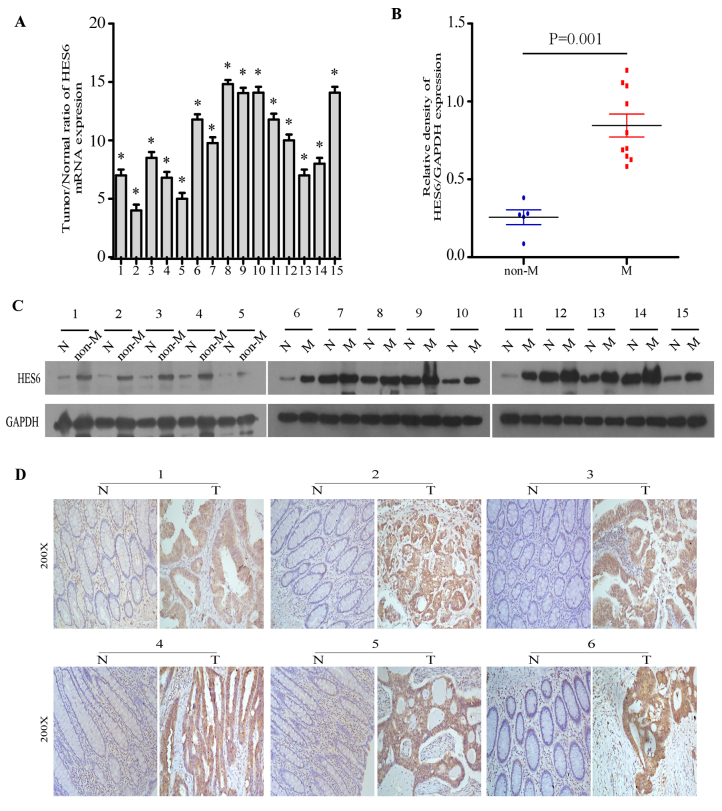
Expression of HES6 in primary human CRC lesions. (A) Average Tumor/Normal ratios of HES6 mRNA expression in paired CRC tissues and adjacent non-cancerous tissues were determined by quantitative polymerase chain reaction and normalized against GAPDH. The error bars represent the standard deviation of the mean calculated from three parallel experiments. (B and C) Western blotting of the expression of HES6 in the adjacent non-cancerous tissues and CRC tissues from primary CRC of 15 patients with or without distant metastasis. GAPDH served as the loading control. (D) The expression of HES6 protein in 6 pairs of matched CRC tissues and adjacent non-cancerous tissues by immunohistochemical analysis. *P<0.05. CRC, colorectal cancer; HES6, hairy and enhancer of split family basic helix-loop-helix transcription factor 6; T, colorectal cancer tissue; N, adjacent non-cancerous tissue; non-M, colorectal cancer tissue without distant metastasis; M, colorectal cancer tissue with distant metastasis.

**Figure 5.**
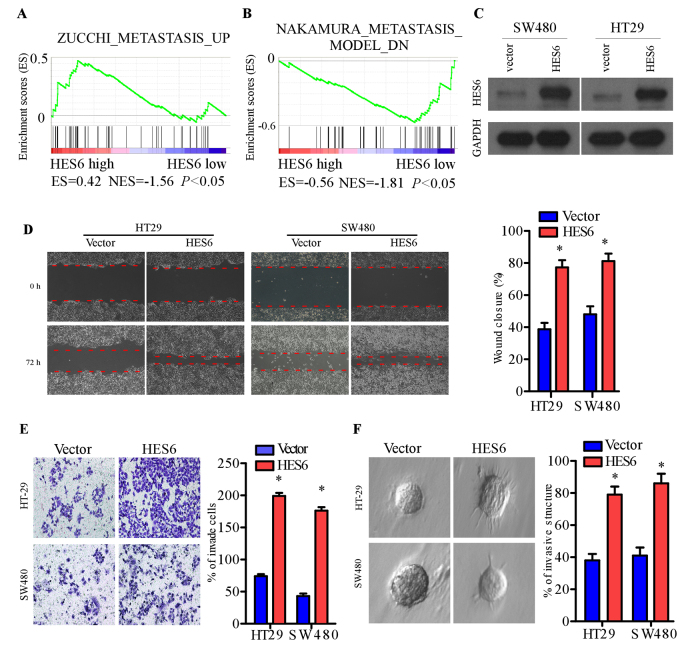
HES6 promotes the migration and invasion of colorectal cancer cells. (A and B) Gene set enrichment analysis plot revealed that HES6 expression was positively correlated with two metastasis gene signatures (ZUCCHI_METASTASIS_UP, NAKAMURA_METASTASIS_MODEL_DN). (C) Western blotting demonstrated overexpression of HES6 in the HT29 and SW480 cell lines. (D) Wound closure by HES6-overexpressing cells in the wound-healing assay. (E) Representative images (left) and quantification (right) of the invasiveness of HES6-overexpressing cells compared with vector control cells in the Transwell matrix invasion assay. (F) Representative images of the invasive structures of HT29 and SW480 cells transduced with vector and HES6 in the three-dimensional spheroid invasion assay. Error bars represent the mean ± standard deviation from three independent experiments. *P<0.05. HES6, hairy and enhancer of split family basic helix-loop-helix transcription factor 6.

